# Medical Journals, Academia, and Industry-Sponsored Clinical Trials

**DOI:** 10.1371/journal.pmed.0020218

**Published:** 2005-07-26

**Authors:** Valeria Frighi

**Affiliations:** **1**Churchill HospitalOxfordUnited Kingdom

Sadly, I fully agree with Richard Smith's opinion [1] that many medical journals have become marketing offices of pharmaceutical companies. Even worse is that few people seem to realize this, and there are many respectable academics who would wholeheartedly dispute these views. The “psychology of gift” operates in every social environment, being so pervasive because it is based on the profoundly human and universal norm of reciprocity [2,3]. In academia and medical publishing it produces great returns to the pharmaceutical industry via clinical scientists who are not dishonest but in a state of denial about their motivations, as Jerome Kassirer, former editor of the *New England Journal of Medicine*, describes very clearly in his recent book [4]. What is happening is also extremely serious because the tainted trials we are offered can make evidence-based medicine a pointless enterprise. Again, something not widely appreciated.

I think Smith's suggestions that there should be more public funding for clinical trials and that journals should critique rather than publish the results of the trials are very interesting. However, how are we going to get publicly, and adequately, funded trials given the current financial climate?

A practical alternative to Smith's suggestion is to play one pharmaceutical company against the other in head-to-head trials, an approach that can help retain independence and that has been used to this purpose before. Another strategy would be for either the regulatory authorities or the academic review boards to demand of the pharmaceutical companies that whenever a new drug is tested in a phase III trial, it should always be done not only against placebo but also against the drug that the condition is generally treated with. This active comparator must be used at the appropriate dose, namely neither too low nor too high (in order to avoid the tested drug spuriously seeming more effective or safe). Moreover, any new licence should be accompanied by a legal requirement for a stringent system of post-marketing surveillance, to be run by the drug company but overviewed by the regulatory authority.

These strategies could possibly help produce results that are more reliable from a scientific point of view, help reduce the number of expensive but not innovative “me too” drugs, and help protect patients' safety more efficiently.

Lastly, I hope Richard Smith will go straight into the lion's den and send his thoughts not only to the similarly minded editors and readers of *PLoS Medicine* but to some of the journals who are the most culpable of the policy he is exposing.

## References

[pmed-0020218-b1] Smith R (2005). Medical journals are an extension of the marketing arm of pharmaceutical companies. PLoS Med.

[pmed-0020218-b2] Cialdini RB (1993). Influence: Science and practice.

[pmed-0020218-b3] Strohmetz DB, Rind B, Fisher R, Lynn M (2002). Sweetening the pill: The use of candy to increase restaurant tipping. J Appl Soc Psychol.

[pmed-0020218-b4] Kassirer JP (2004). On the take: How medicine's complicity with big business can endanger your health.

